# Immune regulation by Tim-3

**DOI:** 10.12688/f1000research.13446.1

**Published:** 2018-03-14

**Authors:** Hridesh Banerjee, Lawrence P. Kane

**Affiliations:** 1 Department of Immunology, University of Pittsburgh School of Medicine, Pittsburgh, PA, 15261, USA

**Keywords:** T cell activation, T cell exhaustion, immunotherapy

## Abstract

T-cell immunoglobulin and mucin domain 3 (Tim-3) is a transmembrane protein that in both mice and humans has been shown to possess various functions in a context-dependent manner. Thus, Tim-3 has been associated with both inhibitory and co-stimulatory function, depending in part on the specific cell type and immune response course. Though originally described on T cells, Tim-3 is now known to be expressed by both lymphoid and non-lymphoid cells within the immune system and even by non-immune cells. In addition, though widely thought of as a negative regulator of immunity, Tim-3 has been shown in more recent studies to have a positive function on both myeloid and lymphoid cells, including T cells. Tim-3 is often expressed at a high level on exhausted T cells in tumors and chronic infection and may engage in crosstalk with other so-called “checkpoint” molecules such as PD-1. Thus, Tim-3 has emerged as a possible therapeutic target, which is being actively explored both pre-clinically and clinically. However, recent research suggests a more complex *in vivo* role for this protein, compared with other targets in this area.

## Introduction

T-cell immunoglobulin and mucin domain 3 (Tim-3) (gene name *Havcr2*) ^1^ is an immunoglobulin (Ig) and mucin domain family cell-surface molecule that was originally identified on CD4 helper 1 (Th1) and CD8 T cytotoxic 1 (Tc1) cells ^2^. Initial studies on the role of Tim-3 in the murine experimental autoimmune encephalomyelitis (EAE) model suggested an inhibitory function on Th1 responses and regulation of macrophage activation and function. Blocking of Tim-3 can lead to the development of spontaneous autoimmunity, at least in some settings ^3^. Corroborating an inhibitory function for Tim-3 is the fact that antibodies against Tim-3 have been shown to enhance anti-viral and anti-tumor T-cell responses, as described below. However, Tim-3 is now known to also be expressed by regulatory T (Treg) cells and innate immune cells such as dendritic cells (DCs), natural killer (NK) cells, monocytes, macrophages, and mast cells. Tim-3 is often referred to as a checkpoint receptor and exhaustion marker; however, Tim-3 has been seen to function differentially, in a context-dependent manner, and now is speculated to have both positive and inhibitory functions ^4– 6^. Here, we will discuss existing evidence for these positive and negative effects of Tim-3 on immune responses and highlight some important unanswered questions.

## Tim-3 structure and ligands

Tim-3 belongs to the Ig super family, with an N-terminal IgV domain, followed by a mucin-like domain that has sites for glycosylation. This is followed by sites for N-linked glycosylation and a single transmembrane domain. The C-terminal cytoplasmic domain does not have any known inhibitory motifs but has five tyrosine residues, two of which have been shown to be phosphorylated and critical for Tim-3-mediated signaling. The IgV domain of Tim-3 consists of two anti-parallel beta sheets that are attached to each other by a disulfide bond. An additional disulfide bond stabilizes the IgV domain and reorients the CC′ loop toward the FG loop, thus forming a unique ligand-binding pocket ^4, 7^.

In humans, shedding of the ecto domain of Tim-3 can take place because of disintegrin and metalloproteases ADAM10 (a disintegrin and metalloprotease 10) and ADAM17. In the absence of the intracellular cytoplasmic domain of Tim-3, this shedding cannot occur, suggesting a role for the cytoplasmic domain of Tim-3 in its cleavage by ADAM10 and ADAM17. Although the relevance of this observation for Tim-3-mediated signaling is not well understood, Tim-3 shedding has been observed with CD14 ^+^ monocytes, in response to lipopolysaccharide stimulation, and in T cells, in the setting of graft-versus-host disease (GVHD) after allogenic hematopoietic cell transplant. Plasma levels of soluble Tim-3 were also found to be elevated in patients with GVHD ^8^. Tim-3 shedding by ADAM10 has also been observed in untreated HIV patients, and this correlates with disease progression ^9^.

At this point, four distinct ligands have been reported to bind to Tim-3 in different contexts. These are galectin-9 (Gal-9), high-mobility group protein B1 (HMGB1), carcinoembryonic antigen cell adhesion molecule 1 (Ceacam-1), and phosphatidylserine (PtdSer). Gal-9 was the first reported ligand for Tim-3 and was shown to induce apoptosis in Th1 cells ^10^, although Gal-9 can bind to other receptors on the cell surface as well ^11^. Interaction of PtdSer with Tim-3 has been shown to play a role in the clearing of apoptotic bodies and also helps in antigenic cross-presentation, although it should be noted that the affinity of PtdSer for Tim-3 is significantly weaker than for other TIM proteins ^12^. Ceacam-1 is the most recently identified ligand for Tim-3 and can form a heterodimer with Tim-3 as well as interacting with Tim-3 *in trans* ^13^. HMGB1 is highly expressed by tumor-infiltrating DCs. In tumors, Tim-3 therefore competes with nucleic acid binding to HMGB1 and lowers the transport of nucleic acids to the endosomes, thereby dampening the innate immune response to tumor-associated nucleic acid ^14^. Liver-primed CD8 ^+^ Tim-3 ^+^ cells were also shown to suppress anti-viral immunity in a Gal-9-independent and HMGB1-dependent manner ^15^. As discussed below, antibodies to Tim-3 are actively being explored as therapeutics. Although these are often referred to as “blocking” antibodies, their ability to block the interaction of Tim-3 with its various ligands is not always documented directly.

## Regulation of Tim-3 expression and function

Transcriptional control of Tim-3 during acute and chronic infection and in tumors is an area of active research. Nuclear factor of activated T cells (NFAT) signaling has been shown to play a role in CD8 ^+^ T-cell regulation of Tim-3 ^16^. T-bet is another transcription factor that has been shown to have a positive effect on Tim-3 expression during T-cell activation ^17^, whereas the same factor appears to have a negative effect in exhausted T cells ^18^. Tim-3 expression has also been shown to be regulated by at least three transcription factors: NFIL3, T-bet, and STAT3. In one report, the authors showed that interleukin-10 (IL-10) and IL-27 together can lead to epigenetic changes in the *Havcr*2 locus ^19^, further supporting the notion that, along with T-cell receptor (TCR) stimulus, cytokines and other extrinsic factors may have differential effects on Tim-3 expression and function.

Tim-3 is generally co-expressed with other checkpoint receptors in settings of T-cell exhaustion in both tumors and chronic infection in both humans and mice ^20^. However, mechanisms by which TCR, and other factors, regulate the expression of Tim-3 during acute versus chronic stimulation are not well defined. Similarly, in tumors, although a significant population of tumor-infiltrating T cells express Tim-3, it is not known what factors in the tumor microenvironment, along with tumor antigen, play a role in the upregulation of Tim-3 on effector and Treg cells.

## Tim-3 signaling

Although initially Tim-3 was found to have an inhibitory function, based on its expression on exhausted T cells and in an autoimmunity model, there is still little direct proof of this concept. There are also some reports which prove that Tim-3 in certain cases may play a co-stimulatory enhancing function. High ectopic expression of Tim-3 on T-cell lines has also been shown to have increased activation based on increased NFAT/AP-1 and nuclear factor-kappa B (NF-κB) reporter assays and enhanced levels of cytokines ^21^ and higher phospho-S6 levels. One possible explanation for contradicting reports with respect to Tim-3 function in T cells relates to the expression of Ceacam-1 along with Tim-3. Thus, it has been suggested that Tim-3 inhibitory function is dependent on the co-expression of Ceacam-1 in both tumors and autoimmune disease ^13^. Another factor which may play an important role, and which complicates the characterization of Tim-3 signaling *in vivo*, is that Tim-3 is also expressed in other cell types.

Multiple tyrosine molecules in the cytoplasmic tail of Tim-3 do not form any recognizable inhibitory motifs. Nonetheless, *in silico* characterization of these sites predicts that they can be substrates for phosphorylation by multiple tyrosine kinases. Indeed, several studies have now shown that tyrosine residues in the cytoplasmic tails of Tim-3 can be phosphorylated ^21, 22^. Both the Src family kinases Fyn and Lck and the Tec family tyrosine kinase Itk have been reported to have a role in Tim-3 cytoplasmic domain phosphorylation. Owing to the involvement of multiple kinases and multiple phosphorylation sites in the Tim-3 cytoplasmic domain, distinct binding of different Tim-3 ligands or antibodies may bring about different outcomes. However, this possibility needs to be explored further.

In support of a possible co-stimulatory function, Tim-3 expression during acute lymphocytic choriomeningitis virus (LCMV) infection is associated with a better short-term effector T-cell response, though possibly at the cost of memory T-cell formation. Furthermore, the absence of Tim-3 leads to defective Akt/mTOR signaling ^23^; however, in the chronic LCMV T-cell exhaustion model, Tim-3 expression was sufficient to dampen the anti-PD-1 rescue of T-cell responses, thereby suggesting crosstalk of PD-1 and Tim-3 in exhausted T cells ^23^, as discussed above. Supporting this finding is the recent report by Gorman and Colgan that acute stimulation in response to LCMV infection leads to upregulation of Tim-3 in persisting Th1-type CD4 cells, and these cells also show enhanced effector functions both *in vitro* and *in vivo* ^24^.

Bat3 is an adapter molecule that has also been shown to act as an inhibitor of Tim-3 signaling by directly binding to the Tim-3 cytoplasmic tail in a Gal-9-reversible manner. Switch of binding of the cytoplasmic domain of Tim-3 from Bat3 to Fyn is speculated to play a role in determining whether Tim-3 signaling positively or negatively affects TCR signaling ^25^. It has also been reported that Tim-3 can co-localize with transmembrane phosphatases such as CD45 and CD148 and that recruitment of Tim-3 to the immunological synapse may lead to destabilization of the synapse and dampening of TCR signaling ^26^. During early pregnancy, in decidual NK cells, Gal-9/Tim-3 signaling has been shown to be important and beneficial for the maintenance of pregnancy ^27^.

## Tim-3 and innate immunity

Tim-3 is known to be expressed on certain innate immune cells, including NK cells, macrophages, DCs, and mast cells. Tim-3 has been found to be expressed by all mature NK cells, and immature NK cells upregulate Tim-3 upon maturation ^28^. Studies on *in vitro* cultured NK cells suggest a co-stimulatory function for Tim-3. In tumors, Tim-3 expression is associated with poor prognosis and suppression of anti-tumor function. Blockade of Tim-3 reverses the exhaustion phenotype of NK cells in certain tumor models ^29, 30^. Tim-3 is constitutively expressed on mast cells and enhances proximal FcεRI signaling, leading to degranulation and cytokine release upon antigen crosslinking, suggesting a co-stimulatory function of Tim-3 in mast cells ^31^.

Tim-3 is expressed on DCs and in tumors was shown to suppress the response to nucleic acid ligands for TLR3, TLR7, and TLR9 and cytosolic sensors to DNA and RNA by impairing HMGB1-mediated recruitment of nucleic acids to endosomes ^14^. In DCs, Tim-3 has also been shown to inhibit activation and maturation via Btk and c-Src to prevent NF-κB signaling ^32^. A recent article also shows that, during chronic HIV infection, Tim-3 may play a role in the dysfunction of plasmacytoid DCs by interfering with TLR signaling via the recruitment of IRF7 and p85 to lysosomes ^33^.

Tim-3 is expressed on macrophages and, in various disease models, has been associated with inhibitory function ^34^. More recently, Tim-3 has been shown to act as a negative regulator of the NLRP3 inflammasome by dampening NF-κB responses in mouse peritoneal macrophages ^35^. The authors further showed that tyrosines 256 and 263 near the Tim-3 C-terminus are necessary for NLRP3 inhibition by Tim-3 and, in a ** model of peritonitis blockade of Tim-3, led to increased pathology. Finally, a recent report also suggests a role for Tim-3 in regulating the resolution of inflammation in an acute lung injury model through effects of Tim-3 ^+^ Treg cells on macrophage polarization ^36^.

## Tim-3 and tumors

Tim-3 is expressed on a significantly higher proportion of tumor-infiltrating lymphocytes compared with its expression in peripheral lymphoid compartments ^1, 37^. Tim-3 upregulation, along with upregulation of other checkpoint receptors, is associated with CD8 T-cell exhaustion. In melanoma, upregulation of Tim-3, along with PD-1, marks a highly non-responsive population of CD8 T cells ^1^. Tim-3 has also been shown to be expressed on tumor antigen-specific T cells in the peripheral blood of patients with various tumors. In mouse models, various types of tumors have been shown to be affected differently in terms of the efficacy of Tim-3 antibody treatment. While there are reports in which anti-Tim-3 antibody treatment did not lead to any inhibition of tumor growth ^38^, there are other studies in which anti-Tim-3 antibody did lead to a slowing of tumor progression by promoting type I anti-tumor immunity ^39^. It has also been reported that Tim-3 ^+^ T cells in head and neck cancer are resistant to PD-1 blockade alone and that there is crosstalk between Tim-3 and PD-1 in CD8 T cells via PI3K/AKT signaling ^40^. It has also been reported that in a head and neck cancer model, increased resistance to cetuximab is associated with increased PD-1 and Tim-3 expression on tumor-infiltrating lymphocytes ^41^, further suggesting the need for a multi-dimensional approach to cancer treatment. Thus, a combination of anti-Tim-3 treatment with other anti-checkpoint receptors and co-receptor receptors is a potentially attractive immunotherapy for cancer. However, it should be noted that how these combination therapies work mechanistically is still under-explored.

In addition to effector T cells, Tim-3 is highly expressed on tumor-infiltrating Treg cells across multiple types of tumors ^42^. A higher frequency of Tim-3 ^+^ tumor-infiltrating lymphocyte Treg cells is also associated with poor patient survival ^37^. Consistent with this, Tim-3 ^+^ tumor Treg cells may be more suppressive than Tim-3 ^−^ Treg cells from the same tumors ^43^. In head and neck tumors, Tim-3 ^+^ Treg cells have also been shown to express higher levels of co-inhibitory molecules such as CTLA-4 and CD39, along with higher levels of FoxP3, CD25, granzyme B, and PD-1. Some tissue-resident Treg cells have also been reported to upregulate Tim-3 ^44^, which has been shown to play a role in tissue homeostasis and repair ^45^, but the exact role of Tim-3 in these tissue Treg cells is still not clear. Treg cells are also known to express Tim-3 during allograft rejection, in which it has been shown that Tim-3-expressing Treg cells are short-lived ^46^. This raises a question about the status of Tim-3-expressing Treg cells in tumors, as it was recently reported that apoptotic Treg cells in the tumor microenvironment may have a more important suppressive function than live Treg cells via an inhibitory effect of ATP on both antigen-presenting cells and effector T cells ^47^.

With regard to non-T cells, Tim-3 is expressed by tumor-associated macrophages in response to tumor-derived factors such as transforming growth factor-beta (TGF-β) ^48^. In addition, Tim-3 low M1 macrophages can upregulate Tim-3 and become M2 macrophages in the tumor microenvironment, directly dampening macrophage function ^49^. It should be noted that a direct role for Tim-3 in M2 macrophages has not been reported. NK cells are also known to play a significant role in tumor clearance, and tumor-associated NK cells constitutively express Tim-3. These NK cells also show an exhausted phenotype, and anti-Tim-3 treatment, as discussed above, leads to the reversal of exhaustion in some tumors. Tim-3 function and regulation in NK tumor immunity are still relatively under-studied, compared with T cells, so future studies may reveal additional layers of Tim-3-mediated crosstalk in tumor immune responses involving NK cells.

There is increasing evidence that Tim-3 can also be expressed by tumor cells themselves ^50– 52^. The expression of Tim-3 on tumor cells may lead to tumor progression by multiple mechanisms, including direct suppression of CD4 T-cell function and inhibition of IL-6–STAT3 signaling and by directly promoting tumor metastasis ^52^. Given the expression of Tim-3 in multiple cell types in different tumor models, it would be very interesting to explore how anti-Tim-3 alone, or in combination with other anti-checkpoint receptor modalities, affects various subpopulations of Tim-3-expressing cells. A more in-depth discussion about the possible roles of Tim-3 in tumors can be found in recent reviews ^53, 54^.

## Tim-3 and pregnancy

A possible role for Tim-3 in pregnancy was first reported by Zhao *et al*., who showed that Tim-3 was upregulated by peripheral blood monocytes (but not T or B cells) ^55^. This group also reported that abnormal Tim-3 levels during pregnancy are associated with pregnancy loss, although it should be noted that a later report suggested that the expression of Tim-3 by peripheral blood CD8 T cells and NK cells was associated with lower cytokine production cytotoxicity ^56^. In pregnant mice, cells from the decidua show high expression of both PD-1 and Tim-3, and these T cells also had higher proliferative capacity and cytokine-producing ability. Treatment of pregnant mice with anti-PD-1 or anti-Tim-3 (or both) leads to pregnant mice becoming highly susceptible to pregnancy loss. The number and function of Tim-3 ^+^PD-1 ^+^ T cells were also seen to be affected in cases of recurrent miscarriage ^57^. In addition, PD-1 and Tim-3 have been shown to induce Th2 bias at the maternal–fetal interface ^58^. The interaction of Gal-9 and Tim-3 was also shown to play a role at the maternal–fetal interface by directly affecting decidual NK cell function ^59, 60^. Furthermore, Tim-3-expressing peripheral NK cells were shown to have immunosuppressive function, resulting in the production of anti-inflammatory cytokines and the induction of Treg cells ^57^. Significantly, in cases of recurrent miscarriage, Tim-3 expression and function on NK cells were found to be defective ^59, 60^. In addition, Tim-3 could protect decidual stromal cells from Toll-like receptor-mediated apoptosis and inflammatory reactions and promotes Th2 bias at the maternal–fetal interface ^58^. Thus, these studies demonstrate that Tim-3 plays important roles in establishing and maintaining the immune-tolerant environment both at the maternal–fetal interface and in the peripheral blood, resulting in successful pregnancy.

## Tim-3 and infectious disease

The role of Tim-3 during infection was first reported in HIV-infected patients, in whom CD8 ^+^ T cells were shown to express Tim-3 and were functionally exhausted ^61^. Tim-3 expression also directly correlated with viral load and was inversely correlated with the use of highly active anti-retroviral therapy. *In vitro* peptide stimulation in the presence of anti-Tim-3 antibody also led to the restoration of cytokine function and proliferation of HIV-specific T cells. Tim-3, along with PD-1, is also highly expressed on mouse and human exhausted T cells in LCMV ^62^, hepatitis B virus ^63^, Friend virus, and hepatitis C virus ^64^ infection. It should be noted that Tim-3, along with PD-1, is associated with terminally exhausted T cells and that in some cases anti-Tim-3, along with anti-PD-L1 antibody, leads to the restoration of cytotoxic function and reduction of viral titer ^62^.

Recent work in our lab highlights an additional role of Tim-3 as a determinant of effector versus memory T-cell differentiation in acute viral infection, consistent with the observation that Tim-3 is rapidly expressed by activated T cells in response to acute LCMV challenge ^23^. This raises an interesting question regarding the possibility of differential regulation and function of Tim-3 in acute versus chronic infection. Thus, in acute infection, there appears to be a co-stimulatory role for Tim-3, leading to differences in mTOR signaling, whereas in the case of chronic infection, Tim-3 marks exhausted cells and may play a direct role in dampening T-cell responses. Alternatively, it is worth considering that the expression of Tim-3 by exhausted T cells represents a last-ditch effort by the immune system to salvage some function from these cells. Further support for a positive role for Tim-3 in driving mTOR activation comes from a recent study of human T cells ^65^.

Consistent with a positive role for Tim-3 in at least some infections, Tim-3 expression by human T cells during *Mycobacterium tuberculosis* (MTb) infection is associated with increased effector function ^66^. For reasons that are unclear, a more recent report, using a mouse model, concluded that Tim-3 helps to maintain T-cell exhaustion on T cells during MTb infection ^67^. The same authors previously reported an indirect mechanism for Tim-3 in both mouse and human MTb infection, whereby Tim-3 expressed by T cells interacts with Gal-9, which in turn stimulated the antibacterial function of macrophages ^68, 69^. Thus, as described elsewhere in this review, significant attention will need to be paid to the effects of Tim-3 on not only T cells but also other cell types that can express this protein. In *Listeria monocytogenes* infection, Tim-3 expression has been shown to correlate positively with an effector phenotype of T cells, and Tim-3 expression has been directly demonstrated to enhance CD8 T-cell responses in this model ^5^.

## Tim-3 and immunotherapy

Establishment of Tim-3 as an exhaustion marker, in both tumors and chronic infection, makes Tim-3 an attractive target for immunotherapy. In mouse tumor models where PD-1 blockade is only partially efficacious, the combination of Tim-3 and PD-1 therapy has been shown to be more effective as a treatment, leading to better tumor regression ^38, 39^. In the case of chronic infection, combined blockade of Tim-3 and PD-1 led to improved CD8 T-cell response and viral control ^62^. Adaptive resistance to PD-1 monotherapy has been associated with the upregulation of other checkpoint receptors and is a current challenge in the field ^70^, including crosstalk between PD-1 and Tim-3 in exhausted/effector T cells ^40^. Combined blockade of Tim-3 along with other checkpoint receptors such as PD-1 and CTLA-4 therefore may be an important therapeutic approach. Long-term protection has also been observed in a murine tumor model when Tim-3 monoclonal antibody was combined with agonist antibodies against the co-stimulatory molecule CD137 on T cells ^71^. Pursuant to a better mechanistic understanding of how anti-Tim-3 antibodies might function in tumor therapy, a recent report showed that previously described antibodies to human or mouse anti-Tim-3 seem to work by blocking the interaction of Tim-3 with PtdSer and Ceacam-1 ^72^.

Recently, the use of chimeric antigen receptor (CAR) T cells was approved by the US Food and Drug Administration as a therapy to treat B-cell lymphoma. Despite some success of CAR T-cell therapy in patients with B-cell acute lymphoblastic leukemia and B-cell malignancies, there is limited efficacy of CAR T cells in solid tumors. One of the probable reported causes of this is the ability of the tumor microenvironment to induce CAR T-cell exhaustion, leading to PD-1 and Tim-3 expression after initial waves of expansion ^56^. It has also been reported that tonic CAR T receptor signaling triggered by antigen-independent clustering can lead to early exhaustion of CAR T cells through the upregulation of Tim-3, PD-1, and LAG-3 ^73^. This further highlights the rationale for exploring anti-Tim-3 therapy, along with other therapeutic approaches, for the treatment of cancer.

Although Tim-3 has been shown to be a promising therapeutic target, a recent report suggests that anti-Tim-3 treatment can lead to more severe inflammation and peribronchiolar fibrosis due to defective clearance of apoptotic bodies ^74^, consistent with the notion that Tim-3 is a receptor for PtdSer on apoptotic cells. Therefore, more detailed and systematic study is needed to determine other potential side effects of anti-Tim-3 antibody treatment.

## Summary

Although Tim-3 was first described as an inhibitory receptor on T cells, it is now known to be expressed by different immune and non-immune cell types. In addition, Tim-3 has now been shown to possess either negative or positive function in various settings, depending on the cell type and physiological or pathological context. All of these findings suggest that current efforts to translate Tim-3 as a target for immunotherapy of cancer will be more complicated than other targets with more limited expression (for example, CTLA-4 and PD-1). Nonetheless, they also indicate that there is more interesting biology surrounding Tim-3 that remains to be deconvolved and that may lead to unanticipated applications for this protein as a therapeutic target.
